# Tracking perceived stress, anxiety, and depression in daily life: a double-downward spiral process

**DOI:** 10.3389/fpsyg.2023.1114332

**Published:** 2023-04-18

**Authors:** Guo Feng, Xiaxia Xu, Jiawei Lei

**Affiliations:** ^1^Psychological Research and Counseling Center, Southwest Jiaotong University, Chengdu, Sichuan, China; ^2^Institute of Applied Psychology, Southwest Jiaotong University, Chengdu, Sichuan, China

**Keywords:** perceived stress, anxiety, depression, experience sampling methodology (ESM), downward spiral model

## Abstract

**Introduction:**

Previous studies using retrospective questionnaires have suggested a complex relationship between perceived stress and related negative emotions and emphasized their importance in mental health. However, how daily perceived stress, anxiety, and depression interact dynamically in a natural context remains largely unexplored.

**Methods:**

This study conducted a longitudinal survey that applied experience sampling methodology to data from 141 Chinese college students (58% women, mean age = 20.1 ± 1.63 years).

**Results:**

The hierarchical linear models confirmed that daily perceived stress and negative emotions (i.e., perceived depression and anxiety) could reciprocally reinforce one another with the characteristic dynamics of a cognitive–emotional downward spiral. Additionally, anxiety and depression could further circularly aggravate each other imminently. These two intertwined downward-spiral processes constitute a double-downward-spiral model.

**Discussion:**

The findings contribute to a better understanding of the interactive mechanisms underlying perceived stress and its related negative emotions in everyday life and highlight the significance of early emotion regulation and stress relief in healthy people.

## Introduction

Negative emotions are a general dimension of subjective distress and unpleasurable activation that subsumes various aversive mood states (Watson et al., 1988). From an evolutionary psychology perspective, negative emotions have adaptive functions, such as helping people identify threats and dangers (Nesse, 1990). In contrast, from the mental health standpoint, negative emotions are more likely to cause a range of dysfunctional reactions and detrimental outcomes, such as impairment of an individual’s thought–activity sequence and chronic burnout of social responsibilities, and predict various forms of psychopathology, particularly mood disorders (Fredrickson and Branigan, 2005; Lapid Pickman et al., 2021; Liu M. et al., 2021). Many studies have proposed that anxiety and perceived depression are the most damaging negative states of mind. They are prevalent in people’s daily lives and lead to serious mental health problems over time without valid interventions (Ren et al., 2016; Monzonís-Carda et al., 2021; Casey et al., 2022).

However, the question remains how seemingly unavoidable negative emotions in daily life gradually deteriorate, resulting in emotional problems or obstacles for healthy individuals. Previous studies, mostly based on cross-sectional and cross-lag longitudinal methodologies, have indicated that perceived stress, anxiety, and perceived depression are potential antecedents of mood disorders (Asmundson et al., 2020; García-Mieres et al., 2020; Armon et al., 2021), and show close associations with each other (Levine et al., 2019; Huang et al., 2021). However, studies have focused only on the effect of major stress events and serious negative emotions, and these variables have rarely been proposed in short-term longitudinal cycles as a potential risk for mood disorders in daily life. In reality, it is the neglected daily trifles that shape our long-term psychological states. For example, daily chores better predict stress-related health issues than major life events (Miller et al., 2008). The accumulation of negative reactions to minor stresses will likely cause health problems and psychological disorders (Miller et al., 2008; Johnsson et al., 2015). Therefore, it is necessary to explore the dynamic internal interaction, mediation, and moderation mechanisms between daily perceived stress and related perceived negative emotions (i.e., anxiety and depression) in a natural context. This study examined a double-downward-spiral process model of daily perceived stress, anxiety, and perceived depression to determine how minor stresses and negative emotions in everyday life gradually become serious and why they lead to mental health problems.

Perceived stress is an individual’s cognitive assessment of the threat posed by stressors and their ability to cope with these threats (Liu Z. et al., 2021). In the process of coping with stressful events, individuals are prone to produce a series of negative emotions such as anxiety, depression, anger, and pain (Pereira-Morales et al., 2019; Huang et al., 2021; Clayborne et al., 2022). For example, Israeli et al. (2018) triggered participants’ perceived stress through an impromptu speech task and found that induced stress promoted a more severe state of anxiety in participants. In addition, a recent experience sampling study also supported that perceived stress at a certain moment in daily life could positively predict imminent anxiety (Du and Xu, 2019). Anxiety can also play a role in perceived stress. Anxiety is often closely related to negative cognitive styles such as rumination (Brosschot et al., 2006), which leads to higher levels of physical and psychological arousal in individuals, causing them to become hyper-focused on future stressors, further exacerbating their subjective experience of stress (Yang and Liu, 2016).

Perceived stress is suggested to predict depression positively. The diathesis–stress theory proposes that specific life stress is essential for triggering depression (Monroe and Simons, 1991). Consistent with this theory, studies have also shown that perceived stress has a stable positive relationship with perceived depression, even in different time windows (Xu et al., 2018; Levine et al., 2019). Individuals in stressful situations may have different evaluation and coping styles to outside stimuli; therefore, they may adopt negative cognitive styles that could worsen depression (Rodríguez-Naranjo and Caño, 2016). However, persistent perceived depression can lead to increased stress. According to the cognitive resource theory (Kahneman, 1973), processing depressed emotions might deplete or divert the resources needed to perform control functions. This diversion of resources leaves individuals with insufficient cognitive resources to cope with environmental events, leading them to perceive more stress (Padmala et al., 2011; Bui et al., 2021). A recent study supported the finding that depressed mothers raising children perceived more parenting stress than healthy mothers (Ma et al., 2019).

Moreover, it is well known that depression and anxiety are highly interdependent, meaning that individuals with high levels of depression (anxiety) tend to have severe anxiety (depression) (Chavira et al., 2004; González-Mesa et al., 2020). Despite the high co-occurrence of depression and anxiety, these disorders differ in nature. According to the tripartite model of anxiety and depression, anxiety is simply a high level of negative emotion, whereas depression combines low levels of positive emotion with high levels of negative emotion. Anxiety is associated with high physical arousal, whereas depression is not (Anderson and Hope, 2008). Thus, in addition to exploring the bidirectional cognitive–emotional facilitation relationship between perceived stress and negative emotions (anxiety and depression), we also examined whether there was mutual reinforcement between anxiety and depression as two distinct emotional states to provide an explanation for their co-occurrence (Kuppens and Verduyn, 2017).

Emotions are not self-generated but are caused by changes in the internal or external (usually social) environment (Kuppens and Verduyn, 2017). Because of constant changes in individuals’ internal and external environments, temporary emotions fluctuate dynamically. Perceived stress is another dynamic variable that can easily change with time, the environment, and other factors (Henderson et al., 2016; Ekuni et al., 2022). However, most previous horizontal and vertical studies have tended to treat stress and negative emotions as relatively “stable” states. Retrospective questionnaires have been widely used to measure stress, anxiety, and depression. However, this approach might have a “memory–experience bias” and fail to sensitively capture the dynamic changes of these variables in daily life because perceived stress and emotions can be situational (Ellison et al., 2020). Therefore, previous results have mostly been based on observations over relatively long periods in different groups. How do individuals’ perceived stress, depression, and anxiety change dynamically and affect each other? This question has not yet been answered well. To answer it, we needed to use a dynamic assessment method to conduct intensive tracking measurements of perceived stress, perceived depression, and anxiety in daily life.

The experience sampling method (ESM) repeatedly collects and records individuals’ instantaneous assessments of daily life in a short period and has been validated by numerous studies (Francis et al., 2021; Parsons and Young, 2022). It can be used effectively to study the trajectory of individual variables over time and context and their related influencing factors. For example, Du et al. (2019) administered five surveys to 100 university students every day for a single week to collect real-time data on their mindful state and positive emotions at each time point. They found that a mindful state and positive emotions could reciprocally enhance each other in a dynamic upward spiral. Compared with retrospective questionnaires, ESM allows us to acquire “immediate” information that reflects participants’ current state and reduces the bias between memory and experience (Ellison et al., 2020). This study used ESM to track individuals’ real-time perceived stress, depression, and anxiety in daily life to explore their mutually reinforcing developmental trajectories because ESM is a realistic and accurate way to draw conclusions generalizable to everyday life.

The above discussion implies that perceived stress, depression, and anxiety can exist in a mutually promoting process, leading to a gradual deterioration in mental health. We refer to this process as a “downward-spiral process of negative emotion.” The downward spiral of stress sensitization theory provides initial evidence to support this process. It has been proposed that daily stressors might not evoke a significant mood episode but instead progressively and covertly lower affective thresholds, leading people to become more sensitive to stress and prone to negative emotions and full-blown episodes of perceived stress and depressive symptoms (Post, 2007). In addition, a previous cross-lag study investigated the downward-spiral model between perceived stress and depressive symptoms. Levine et al. (2019) tested 658 first-year students using stress, depressive, and perfectionism questionnaires four times every 2–3 months. The results showed that freshmen experienced increased stress and depressive symptoms that contributed to each other longitudinally in a circular and additive manner; this was moderated by self-critical perfectionism (Levine et al., 2019). However, few studies have used a more sensitive and realistic method, such as ESM, to explore the interaction effect model of perceived stress, anxiety, and depression in healthy people’s daily lives. Therefore, we constructed and examined a double-downward-spiral model with two parts: (1) a cognitive–emotional downward-spiral process of perceived stress and negative emotions (anxiety and depression) and (2) an emotional–emotional downward-spiral process of anxiety and perceived depression.

This study aimed to provide evidence of the dynamic reciprocal or mediating link between perceived stress, anxiety, and depression in normal daily life. We conducted ESM study to provide new insights into the dynamically perceived stress, anxiety, and depression experienced by individuals over time in diverse everyday contexts. Our hypotheses were as follows:

H1: Perceived stress at time *t* + 1 mediates the positive prediction of negative emotions (i.e., anxiety and depression) at time *t* on negative emotions (i.e., anxiety and depression) at time *t* + 2, and, in turn, negative emotions (i.e., anxiety and depression) at time *t* + 1 mediate between perceived stress at time *t* and time *t* + 2. This is an examination of the cognitive–emotional downward-spiral model.
H2: Anxiety at time *t* predicts higher anxiety at time *t* + 2 through more perceived depression at time *t* + 1 and vice versa, and perceived depression at time *t* predicts perceived depression at time *t* + 2 through more anxiety at time *t* + 1. This is an examination of the emotion–emotion downward-spiral model.

**FIGURE 1:**
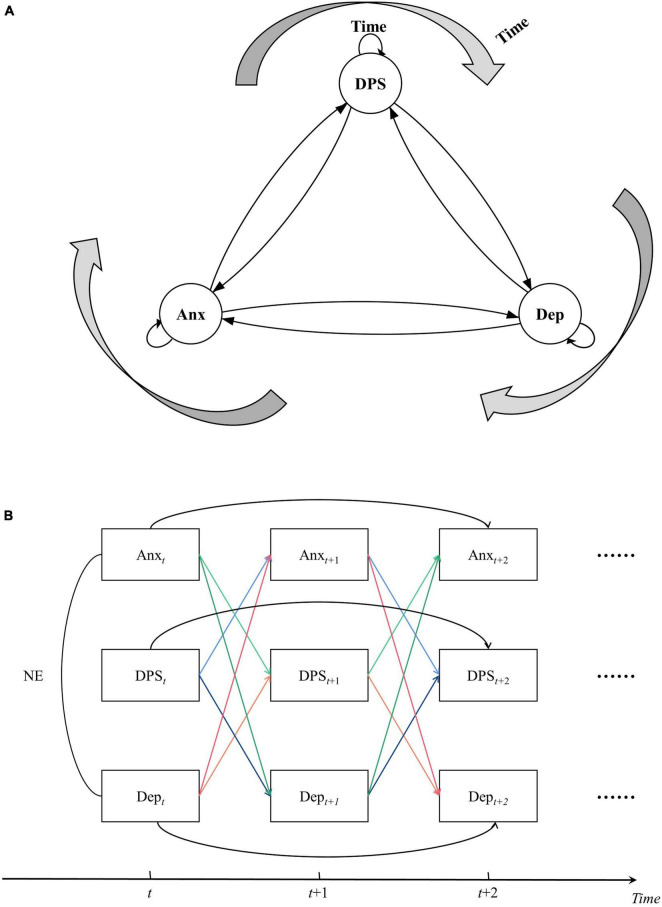
**(A)** A downward-spiral model, presenting a trajectory of change of daily perceived stress, anxiety and depression. DPS, daily perceived stress; Anx, anxiety; Dep, depression. **(B)** The paths of the downward spiral model. DPSt, daily perceived stress at *t*; Anxt, anxiety at *t*; Dept, depression at *t*; NE, negative emotion.

Figures 1A, B illustrate the theoretical double-downward-spiral model.

## Materials and methods

### Participants and procedures

We recruited all the study participants through social media (WeChat and QQ, two popular communication platforms in China) and posters at a Chinese university. The initial sample consisted of 172 participants, but we excluded 31 participants with completion rates below 75%. The final sample comprised 141 students (58% women and 42% men) with a mean age of 20.1 years (*SD* = 1.63) (except for five participants for whom demographic information was unavailable).

First, we invited all the participants to join a group on the social networking platform WeChat, informing them of the research details and securing written informed consent. Over the next 12 consecutive days (19–30 October 2020), they had to complete a brief questionnaire three times daily (at 9:30, 15:30, and 22:30), assessing their perceived stress, subjective depression, and anxiety levels. Thus, each participant completed 36 questionnaires. According to Bolger et al.’s (2012) power simulations curve, power is improved more by increasing sample size than time points, and a power of 80% can be achieved with under 120 samples while keeping the time points to nine. Our study’s sample size was 141, and the number of time points was 36. Therefore, our study’s power was suitable.

The participants accessed the questionnaire each time by responding to a web link we sent to their cell phones via WeChat. We asked all the participants to allow new message notifications from WeChat and to complete the questionnaire within one hour of receiving the link. We offered each participant RMB2–3 (about US$ 0.30–0.45) per completed questionnaire. The university’s ethics committee approved this study, and all participants declared they had no history of mental disorders.

### Measures

#### Daily perceived stress (DPS)

Following previous experience sampling studies (Xu et al., 2018), we used three items to measure perceived stress, all derived from the Perceived Stress Scale (Cohen et al., 1983): (1) feeling unable to control important things in one’s life; (2) feeling nervous and stressed; and (3) constantly thinking that things must be done by oneself. The rating score ranged from 0 (not at all) to 100 (extremely). The higher the mean score for these three items, the greater the perceived stress. The Cronbach’s alpha for the study sample was 0.83.

#### Perceived depression and anxiety

Many previous studies have used dynamic assessment methods to test emotions, primarily through single self-scripted items whose effectiveness has been verified (Xu et al., 2018; Pawluk et al., 2021). We used a single self-administered questionnaire to measure negative emotions (i.e., perceived anxiety and depression). Referring to a previous study (Xu et al., 2018), we used the specifically compiled item “Are you anxious at this moment” to measure perceived anxiety and the item “Are you depressed at the moment” to measure perceived depression. The rating score ranged from 0 (not at all) to 100 (extremely), with higher scores indicating more severe anxiety or depression. We also used the hierarchical linear model (HLM 7.0) to analyze the reliability estimates for anxiety and perceived depression. The reliability estimates for anxiety and perceived depression were 0.970 and 0.975, respectively.

### Analysis using hierarchical linear models

Because we needed to examine both the relationships within individuals and between individuals, we used HLM7.0 to construct a 1-1-1 mediated hierarchical linear model since all variables in the model were measured at Level 1 (within individuals). Following Du and Xu (2019), we tested the mediation process via CWC(M) (centered within the context of the reintroduction of the subtracted means at Level 2) (Zhang et al., 2009) following these analysis steps:

**Step 1.** We established an equation between the independent variable (negative emotions at *t*) and the dependent variable (negative emotions at *t* + 2) in Level 1. We calculated the average stress measured by each participant over 12 days and added that to Level 2 to create Model 1:

MODEL 1

Level 1:


$$N\,e\,g_{\left(t+2\right)\,i}=\pi_{0\,i}+\pi_{1\,i}*\left(N\,e\,g_{t\,i}\right)+e_{t\,i}$$

Level 2:


π0⁢i=b00+b01*(Mi-⁢N⁢e⁢g)+u0⁢i

$$\pi_{1\,i}=b_{10}$$

where *t* represents the moment, and *i* represents the individual. Neg*_(t +_*_2_*_)*i*_* represents the negative emotions of the *i*th participant at *t +* 2 and Neg*_(t)*i*_* represents the perceived stress of the *i*th participant at *t*. M*_*i*_*_Neg is the average of all negative emotions measured by the *i*th participant, which represents the total negative emotions of the participant. The intercept π_0_*_*i*_* represents the mean value of negative emotions measured by the *i*th participant. *e*_*ti*_ denotes the residual. *b*_10_ represents the predictive degree of negative emotions at *t* to negative emotions at *t +* 2 and *b*_01_ represents the predictive effect of overall negative emotions on negative emotions at *t +* 2.

**Step 2.** We established the relationship between the independent variable (negative emotions at *t*) and mediating variable (daily perceived stress at *t* + 1) at Level 1. Then, the negative emotions of each individual are averaged and put into Level 2 in the equation, as follows:

MODEL 2

Level 1:


$$D\,P\,S_{\left(t+1\right)\,i}=\pi_{0\,i}+\pi_{1\,i}*\left(N\,e\,g_{t\,i}\right)+e_{t\,i}$$

Level 2:


$$\pi_{0\,i}=b_{00}+b_{01}*\left(M_{i}\,\_\,N\,e\,g\right)+u_{0\,i}$$

$$\pi_{1\,i}=b_{10}$$

Similar to the first equation, DPS*_(t +_*_1_*_)i_* represents the *i*th participants’ daily perceived stress at *t +* 1. In this model, *b*_10_ represents the prediction degree of negative emotions at one moment to daily perceived stress at the next moment, and *b*_01_ represents the predictive effect of overall negative emotions on daily perceived stress at *t +* 1.

**Step 3.** We tested the relationship between the independent variable (negative emotions at *t*), mediating variable (daily perceived stress at *t* + 1), and dependent variable (negative emotions at *t +* 2) at Level 1. The means of the independent variable (negative emotions at *t*) and mediating variable (daily perceived stress at *t* + 1) for each participant were calculated and placed in Level 2, as seen in Model 3.

MODEL 3

Level 1:


$$N\,e\,g_{\left(t+2\right)\,i}=\pi_{0\,i}+\pi_{1\,i}*\left(D\,P\,S_{\left(t+1\right)\,i}\right)+\pi_{2\,i}*\left(N\,e\,g_{t\,i}\right)+e_{t\,i}$$

Level 2:


$$\pi_{0\,i}=b_{00}+b_{01}*\left(M_{i}\,\text{\_}\,D\,P\,S\right)+b_{02}*\left(M_{i}\,\text{\_}\,N\,e\,g\right)+u_{0\,i}\,I$$

$$\pi_{1\,i}=b_{10}$$

$$\pi_{2\,i}=b_{20}$$

Many of the symbols in this equation are similar to those above. Mi_DPS, which has not appeared before, represents all participants’ overall daily perceived stress. In this equation, *b*_10_ and *b*_20_ represent the predictive effect of negative emotions at t and daily perceived stress at *t* + 1 on negative emotions at *t* + 2, respectively, and *b*_01_ and *b*_02_ represent the extent to which overall negative emotions and daily perceived stress could predict negative emotions at *t* + 2.

Using the same procedure described above, we analyzed the mediation model among daily perceived stress at *t* (the independent variable), negative emotions at *t* + 1 (the mediating variables), and daily perceived stress at *t* + 2 (the dependent variable). As above, we analyzed the mediation effect among anxiety at *t* (the independent variable), perceived depression at *t* + 1 (the mediating variable), and anxiety at *t* + 2 (the dependent variable). Then, we examined the mediating role of anxiety at *t* + 1 in the dynamic association between perceived depression at t and perceived depression at *t* + 2.

## Results

We excluded 31 participants with completion rates below 75%, leaving us with 4,015 valid responses from 141 participants (>75% completion) for analysis. The excluded participants’ stress and anxiety assessments did not differ significantly from those of the included participants (*t*_*stress*_ = –0.640, *p* = 0.523; *t*_*anxiety*_ = –0.292, *p* = 0.771); however, their perceived depression levels were significantly higher than those of the included participants (*t*_*depression*_ = 2.411, *p* = 0.017).

Each included participant responded 28.48 times on average. We deleted missing data at Level 1 when making an MDM file in HLM 7.0. We used the Level 1 data to examine the temporal association among the variables. Table 1 shows the primary variables, including means, standard deviations, and intraclass correlation coefficients (ICCs). ICC (1) is the ratio of between-group variance to the total variance, and ICC (2) is the group mean reliability (Castro, 2002). The null models showed that the ICC (1) values of daily perceived stress, depression, and anxiety were 0.685, 0.606, and 0.566, respectively, indicating that 68.5, 60.6, and 56.6% of the variances were due to inter-individual factors, and 31.5, 39.4, and 43.4% were due to intra-individual factors. The ICC (2) values were all greater than 0.70, indicating that the multilayer linear model was appropriate for this study (LeBreton and Senter, 2008).

**TABLE 1:** Descriptive statistics: means, standard deviations, and ICCs.

Variables	*N*	Mean	*SD*	*ICC(1*)	*ICC(2*)
Daily perceived stress_(_*_*t*_*_+ 2)_	4015	37.64	25.07	0.685	0.984
Depression_(_*_*t*_*_+ 2)_	4015	15.27	21.96	0.606	0.977
Anxiety_(_*_*t*_*_+ 2)_	4015	32.78	28.08	0.566	0.973

### Cognitive–emotional downward-spiral model

This study explored the interconnections between perceived stress, anxiety, and depression in healthy individuals’ everyday lives. Therefore, we first examined the relationships within and between individuals with perceived stress and negative emotions (perceived anxiety and depression).

In the mediating model of negative emotions at *t*, daily perceived stress at *t* + 1, and negative emotions at *t* + 2 (results shown in Tables 2, 3), we found that a higher level of participants’ negative emotions at *t* predicted more negative emotions at *t* + 2 (*b*_10(Anxiety(t)→*Anxiety(t* + 2)) =_ 0.146, *SE* = 0.037, *p* < 0.001; *b*_10(Depression(t)→*Depression(t* + 2)) =_ 0.121, *SE* = 0.034, *p* < 0.001). Similarly, Level 1 results showed that negative emotions at *t* were significantly positively correlated with daily perceived stress at *t* + 1 (*b*_10(Anxiety(t)→*DPS(t* + 1)) =_ 0.229, *SE* = 0.026, *p* < 0.001; *b*_10(Depression(t)→*DPS(t* + 1)) =_ 0.168, *SE* = 0.033, *p* < 0.001). Moreover, this mediating model revealed that daily perceived stress at *t* + 1 played a remarkable mediating role in the association between negative emotions at t and *t* + 2 (*b*_10(DPS(t + 1)→*Anxiety(t* + 2)) =_ 0.260, *SE* = 0.036, *p* < 0.001; *b*_10(DPS(t + 1)→*Depression(t* + 2)) =_ 0.072, *SE* = 0.028, *p* < 0.05).

**TABLE 2:** Step 1 and Step 3 in mediating analysis of Anxiety*_*t*_*, DPS*_*t*_*_+ 1_, and Anxiety*_*t* +_*_2_ are outside the parentheses; mediating analysis of Depression*_*t*_*, DPS*_*t* +_*_1_, and Depression*_*t* +_*_2_ are inside the parentheses.

Negative emotion (dependent variable)	Step 1. Independent variable			Step 3. Independent variableand mediating variable					
	**Anxiety*_*t*_* (Depression*_*t*_*)**			**Anxiety*_*t*_* (Depression*_*t*_*)**			**DPS *_*t* +_*_1_**		
	***B***	***SE***	***p***	***B***	***SE***	***p***	***B***	***SE***	***p***
Anxiety*_*t*_*_+ 2_ (Depression*_*t*_*_+ 2_)	0.146 (0.121)	0.037 (0.034)	< 0.001 (<0.001)	0.086 (0.109)	0.028 (0.035)	< 0.01 (<0.01)	0.260 (0.072)	0.036 (0.028)	< 0.001 (<0.05)

**TABLE 3:** Step 2 in mediating analysis of Anxiety*_*t*_*, DPS*_*t*_*_+ 1_, and Anxiety*_*t* +_*_2_ and mediating analysis of Depression*_*t*_*, DPS*_*t* +_*_1_, and Depression*_*t* +_*_2_.

Step 2. Independent variable	DPS*_*t*_*_+ 1_ (Mediating variable)		
	***B***	***SE***	***p***
Anxiety*_*t*_*	0.229	0.026	<0.001
Depression*_*t*_*	0.168	0.033	<0.001

We also examined the mediating model of daily perceived stress at *t*, negative emotions at *t* + 1, and perceived stress at *t* + 2 (results shown in Tables 4, 5). The model indicated that participants’ daily perceived stress at *t* could predict higher perceived stress at *t* + 2 (*b*_10(DPS(t)→DPS(t + 2)) =_ 0.255, *SE* = 0.030, *p* < 0.001). Our results furtherly demonstrated that daily perceived stress at *t* was positively associated with negative emotions at *t* + 1 (*b*_10(DPS(t)→Anxiety(t + 1)) =_ 0.298, *SE* = 0.043, *p* < 0.001; *b*_10(DPS(t)→Depression(t + 1)) =_ 0.108, *SE* = 0.028, *p* < 0.001). We also found negative emotions at *t* + 1 were remarkably positively related to daily perceived stress at *t* + 2 after controlling for daily perceived stress at *t* (*b*_10(Anxiety(t + 1)→DPS(t + 2)) =_ 0.255, *SE* = 0.030, *p* < 0.001; *b*_10(Depression(t + 1)→DPS(t + 2)) =_ 0.255, *SE* = 0.030, *p* < 0.001). Notably, the dynamic relationship between daily perceived stress at *t* and *t* + 2 was also significantly mediated by perceived depression and anxiety at *t* + 1.

**TABLE 4:** Step 1 and Step 3 in mediating analysis of DPS*_*t*,_* Anxiety*_*t* +_*_1,_ and DPS*_*t*_*_+ 2_ and mediating analysis of DPS*_*t*,_* Depression*_*t* +_*_1,_ and DPS*_*t*_*_+ 2_.

Dependent variable	Step 1. Independent variable			Step 3. Independent variable and mediating variable											
	**DPS*_*t*_***			**DPS*_*t*_→*Anxiety*_*t* +_*_1_→DPS*_*t*_*_+ 2_**						**DPS*_*t*_→*Depression*_*t* +_*_1_→DPS*_*t*_*_+ 2_**					
				**DPS*_*t*_***			**Anxiety*_*t* +_*_1_**			**DPS*_*t*_***			**Depression*_*t* +_*_1_**		
	***B***	***SE***	***p***	***B***	***SE***	***p***	***B***	***SE***	***p***	***B***	***SE***	***p***	***B***	***SE***	***p***
DPS*_*t*_*_+ 2_	0.255	0.030	< 0.001	0.203	0.024	<0.001	0.174	0.022	<0.001	0.240	0.028	<0.001	0.135	0.027	<0.001

**TABLE 5:** Step 2 in mediating analysis of DPS*_*t*,_* Anxiety*_*t* +_*_1,_ and DPS*_*t*_*_+ 2_ and mediating analysis of DPS*_*t*,_* Depression*_*t* +_*_1,_ and DPS*_*t*_*_+ 2_.

Step 2. Independent variable	Mediating variable					
	**Anxiety*_*t* +_*_1_**			**Depression*_*t* +_*_1_**		
	***B***	***SE***	***p***	***B***	***SE***	***p***
DPS*_*t*_*	0.298	0.043	<0.001	0.108	0.028	<0.001

### Emotional–emotional downward-spiral model

We also explored the dynamic relationship and influence of the trajectory between perceived anxiety and depression. The results showed that perceived depression at *t* + 1 was an effective mediator for the dynamic relationship between anxiety at *t* and anxiety at *t +* 2 (*b*_10(Anxiety(t)→Depression(t + 1))_ = 0.102, *SE* = 0.021; *b*_10(Depression(t + 1)→Anxiety(t + 2))_ = 0.118, *SE* = 0.031; *p*s < 0.001). However, the results also showed that anxiety at *t* + 1 played a remarkable mediating role in the dynamic relationship between perceived depression at *t* and perceived depression at t + 2 (*b*_10(Depression(t)→Anxiety(t + 1))_ = 0.155, *SE* = 0.037; *b*_10(Anxiety(t + 1)→Depression(t + 2))_ = 0.080, *SE* = 0.012; *p*s < 0.001). Tables 6, 7 present the results.

**TABLE 6:** Step 1 and Step 3 in mediating analysis of Depression*_*t*,_* Anxiety*_*t* +_*_1,_ and Depression*_*t*_*_+ 2_ are outside the parentheses; mediating analysis of Anxiety*_*t*,_* Depression*_*t* +_*_1,_ and Anxiety*_*t*_*_+ 2_ are inside the parentheses.

Dependent Variable	Step 1. Independent variable			Step 3. Independent variable and mediating variable					
	**Depression*_*t*_* (Anxiety*_*t*_*)**			**Depression*_*t*_* (Anxiety*_*t*_*)**			**Anxiety*_*t* +_*_1_ (Depression*_*t* +_*_1_)**		
	***B***	***SE***	***p***	***B***	***SE***	***p***	***B***	***SE***	***p***
Depression*_*t*_*_+ 2_ (Anxiety*_*t*_*_+ 2_)	0.121 (0.146)	0.034 (0.037)	<0.001 (<0.001)	0.109 (0.134)	0.015 (0.034)	<0.01 (<0.001)	0.080 (0.118)	0.012 (0.031)	<0.001 (<0.001)

**TABLE 7:** Step 2 in mediating analysis of Depression*_*t*,_* Anxiety*_*t* +_*_1_, and Depression*_*t*_*_+ 2_ are outside the parentheses; mediating analysis of Anxiety*_*t*,_* Depression*_*t* +_*_1_ and Anxiety*_*t*_*_+ 2_ are inside the parentheses.

Step 2. Independent variable	Mediating variable		
	**Anxiety*_*t* +_*_1_ (Depression*_*t* + 1_*)**		
	***B***	***SE***	***p***
Depression*_*t*_* (Anxiety*_*t*_*)	0.155 (0.102)	0.037 (0.021)	<0.001 (<0.001)

## Discussion

This research aimed to determine how natural stresses and negative emotions (i.e., anxiety and perceived depression) dynamically interact in everyday life and why they can gradually become severe and lead to mental health problems. Therefore, we examined a double-downward-spiral process model of daily perceived stress, anxiety, and depression using an ESM approach. Consistent with our initial hypothesis, the results showed that perceived anxiety or depression at *t* predicted a higher level of perceived stress at *t* + 1, whereas perceived stress at *t* + 1 predicted more severe perceived anxiety or depression at *t* + 2. These results suggest that each time participants perceived more anxiety and depression, they perceived higher stress at the next moment and then experienced still more perceived anxiety and depression. In other words, the hysteretic positive prediction of the same negative emotion was dynamically mediated by perceived stress. Additionally, perceived stress at *t* predicted a higher level of perceived stress at *t* + 2 via an increase in perceived anxiety or depression at *t* + 1. These findings suggest that daily perceived stress and negative emotions (i.e., anxiety and perceived depression) could mutually and reciprocally reinforce one another with the characteristic dynamics of a cognitive–emotional downward spiral (Hypothesis 1) in healthy individuals’ daily lives. People perceive stress and negative emotions every day; these emotions reinforce each other and become increasingly negative if they do not regulate or handle them properly.

The results also supported the emotional–emotional downward-spiral model of Hypothesis 2 between perceived anxiety and depression. That is, perceived anxiety at this moment would lead to more severe depression in the next moment, and then more severe depression would cause more anxiety in a lagging moment; subsequently, the expanded anxiety would further promote later perceived depression. These two intertwined downward-spiral processes constitute a double-downward-spiral model. Our results corroborated and extended the findings of many previous studies on the association between perceived stress, anxiety, and depression.

In the cognitive–emotional downward-spiral process, perceived stress and negative emotions appeared to be reciprocally strengthened. This finding aligned with the cognitive model of depression. Beck (2008) comprehensively reviewed the psychological and biological correlates of depression and deemed that stress can trigger depression and is probably mediated by cognitive distortions. From the perspective of cognitive resource allocation, daily perceived stress might encourage individuals to be highly alert and induce negative emotions (Bulley et al., 2017). Negative emotions might also largely occupy attentional resources and prevent individuals from focusing on information more conducive to solving problems, resulting in more passive and fixed thinking (Meinhardt and Pekrun, 2003). Negative emotions associated with threats might induce rumination (Curci et al., 2013), which can impair the analytical thinking and executive control needed to cope with stressful life events and lead to inefficient processing and increased self-criticism as individuals further perceive the stress of unresolved life events (Bernstein et al., 2017; Zuroff et al., 2021). This cognitive–emotional downward-spiral process is essential in understanding cognitive–emotional interactions in theory (Simpson et al., 2001). Symptomatology does not occur in isolation; examining how stress and negative emotions influence each other helps us understand the effect of these variables on mental health and how poor mental health outcomes could be sustained over time. In practice, these results imply that an effective way to prevent mental problems would be to interrupt the downward spiral of perceived stress and negative emotions (anxiety and depression) in daily life.

Our results also demonstrated an emotional–emotional downward spiral of perceived anxiety and depression in everyday contexts. More anxiety might expose individuals to more depression later; in turn, individuals who experience more depression might be more anxious at the next moment. Empirical research supports our results. For instance, studies have found that anxious individuals tend to experience persistent negative rumination (Modini et al., 2018), which leads to fatigue (Querstret and Cropley, 2012). Fatigued individuals exhibit more depressive symptoms than those who are not fatigued (Corfield et al., 2016). Studies have also suggested that individuals with depression have lower self-efficacy and are more likely to perceive anxiety when facing external events (Dai and Feng, 2008; Sharma and Kumra, 2022). This finding provides further insight into understanding and extending the self-predictive emotion system enabling individuals to respond directly to their emotions rather than uncontrolled ongoing events (Kuppens and Verduyn, 2015).

Prior studies on the relationship between stress, anxiety, and depression have mostly focused on participants who met those clinical diagnostic criteria (Price et al., 2019; Tolin et al., 2021; Truchot et al., 2021). However, daily prevention is more practical and effective than treatment alone. Addressing the dynamic processes of emotions and their regulation in healthy people has great value (Kuppens and Verduyn, 2015). The current study found that the interconnection of perceived stress, anxiety, and depression among typical college students followed a dynamic downward spiral that increased their perceived stress, anxiety, and depression over time. College students daily stressors include academic performance, interpersonal relationships, life adjustment, and employment (Li and Mei, 2002). Without effective regulation of the stress triggered by these daily pressures, long-term maintenance and accumulation of perceived stress and induced negative emotions can synergistically lead to serious mood disorders (Kong et al., 2021). Therefore, action is necessary to interrupt the downward spiral and mitigate the deterioration of negative cognitions and emotions to prevent mood disorders. In clinical practice, interventions for established mood disorders require more therapeutic resources and time than interventions for negative cognition and emotions in daily life. Therefore, daily interventions are of practical importance for maintaining emotional health as a low-cost, high-yield way to prevent psychological problems (Beames et al., 2021).

Although this study produced some innovative results, it had some limitations. First, emotions include three dimensions: physiological arousal, subjective experience, and behavioral responses (Kuppens and Verduyn, 2017). However, we only examined the subjective experience of emotions and not physiological arousal or behavior. Future studies should use all three dimensions to comprehensively and dynamically assess daily stress and negative emotions. Second, our results were based on a sample of college students in China; thus, they should be applied cautiously to other populations. Third, we did not consider specific natural factors that might confuse the double-downward-spiral processes. Future studies should consider more contextual mediating or moderating factors, such as positive emotions, which might have a potential buffering effect on this downward-spiral process. Also, the dropout participants showed higher perceived depression than the participants who completed more than 75% of the tests, implying that the depressive mood affected their daily life behavior and highlighting the need for more research considering the dynamic negative emotions of daily life.

In conclusion, this study provides further insight into the dynamic interaction between perceived stress, anxiety, and depression in an everyday context through an ESM investigation. We found that daily perceived stress and negative emotions (i.e., perceived anxiety and depression) could reinforce one another over time in a cognitive–emotional downward spiral; also, perceived anxiety and depression could also affect each other over time by activating an emotional–emotional downward spiral. These results imply a double-downward-spiral process model to better understand the reciprocal relationship of daily perceived stress, anxiety, and perceived depression in a natural context. Our findings highlight the value of timely regulation and intervention of subtle stress and negative emotions in daily life and extend the results of previous work on the associations between stress and negative emotions.

## Data availability statement

The raw data supporting the conclusions of this article will be made available by the authors, without undue reservation.

## Ethics statement

The studies involving human participants were reviewed and approved by the Ethics Committee of Southwest Jiaotong University. The patients/participants provided their written informed consent to participate in this study.

## Author contributions

GF involved in the conceptualization of the study, writing, revising, and editing the manuscript, and the supervision of the project. XX involved in collecting the data, writing the manuscript, and data analyses. JL involved in collecting the data and reviewing the manuscript. All authors have read and agreed to the published version of the manuscript.
